# Development of Query Strategies to Identify a Histologic Lymphoma Subtype in a Large Linked Database System

**Published:** 2007-05-04

**Authors:** Michael Graiser, Susan G. Moore, Rochelle Victor, Ashley Hilliard, Leroy Hill, Michael S. Keehan, Christopher R. Flowers

**Affiliations:** 1 Emory University School of Medicine, Winship Cancer Institute, Oncology Informatics, 1365 Clifton Road, N.E., Atlanta, GA, U.S.A; 2 NuTec Health Systems, LaGrange, TX, U.S.A

**Keywords:** Large linked database, cancer outcomes research, cancer epidemiology, cancer registry

## Abstract

**Background:**

Large linked databases (LLDB) represent a novel resource for cancer outcomes research. However, accurate means of identifying a patient population of interest within these LLDBs can be challenging. Our research group developed a fully integrated platform that provides a means of combining independent legacy databases into a single cancer-focused LLDB system. We compared the sensitivity and specificity of several SQL-based query strategies for identifying a histologic lymphoma subtype in this LLDB to determine the most accurate legacy data source for identifying a specific cancer patient population.

**Methods:**

Query strategies were developed to identify patients with follicular lymphoma from a LLDB of cancer registry data, electronic medical records (EMR), laboratory, administrative, pharmacy, and other clinical data. Queries were performed using common diagnostic codes (ICD-9), cancer registry histology codes (ICD-O), and text searches of EMRs. We reviewed medical records and pathology reports to confirm each diagnosis and calculated the sensitivity and specificity for each query strategy.

**Results:**

Together the queries identified 1538 potential cases of follicular lymphoma. Review of pathology and other medical reports confirmed 415 cases of follicular lymphoma, 300 pathology-verified and 115 verified from other medical reports. The query using ICD-O codes was highly specific (96%). Queries using text strings varied in sensitivity (range 7–92%) and specificity (range 86–99%). Queries using ICD-9 codes were both less sensitive (34–44%) and specific (35–87%).

**Conclusions:**

Queries of linked-cancer databases that include cancer registry data should utilize ICD-O codes or employ structured free-text searches to identify patient populations with a precise histologic diagnosis.

## Background

Linking legacy clinical and administrative databases provides a novel resource for investigating cancer risk factors and predictors of clinical outcomes. However, using large linked databases (LLDB) for medical research purposes is limited by several factors. First, reliance upon coded outcomes such as International Classification of Diseases, Ninth Revision (ICD-9) diagnosis codes can lead to significant inaccuracies (Benesch et al. 1997; Guevara et al. 1999; Rosamond et al. 2004; Verstraeten et al. 2003). ICD-9 provides a classification system for assigning codes to diagnoses and procedures associated with healthcare utilization, but frequently are assigned by personnel unfamiliar with the patient, disease or procedure being coded. Second, the use of patient identifiers such as social security numbers to link data across heterogeneous databases can lead to data integrity problems caused by data entry errors, incomplete data entry, or inconsistent practices such as entering a mother’s social security number for a child whose identifier is not available (Graiser et al. 2005d). Third, some LLDBs capture identical data points from multiple sources which compound the inaccuracies unique to each data source. Users of query tools for searching LLDBs need the most effective search strategies for identifying relevant information, if these data are to be used to perform meaningful clinical and epidemiological research (Koroukian et al. 2003; McClish et al. 1997; Warren and Harlan, 2003; Benesch et al. 1997).

**Figure 1.**
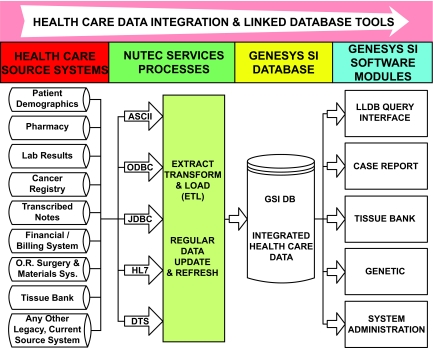
System architecture for the GeneSys SI oncology database application

GeneSys SI represents a LLDB that is a fully integrated platform combining clinical, administrative, and genetic databases to allow researchers to simultaneously query multiple source databases and therefore facilitate cancer outcomes research (Graiser et al. 2005b). Rather than replacing existing databases and systems, this platform is designed to interface with an institution’s existing databases to create a stand-alone SQL-based, data warehouse that can be readily accessed by researchers. GeneSys SI was jointly developed through a partnership between Emory University’s Winship Cancer Institute and NuTec Health Systems to link data for 180,000 oncology patients including data from legacy administrative (Health-Quest: hospital; IDX: clinic), cancer registry (IMPAC Medical Systems), electronic medical records (Cerner PowerChart), laboratory, pharmacy, clinical trials databases as well as newly developed genomics and microarray databases. The source systems feeding the LLDB independently store the following diagnosis data: cancer registry International Classification of Diseases for Oncology (ICD-O) topography and histology codes (a SEER standard format), three sources of ICD-9 diagnosis codes from the hospital, clinic, and radiation oncology, and electronic medical record reports such a physician notes and pathology reports. A summary of data sources is shown in Table 1. The system architecture is illustrated in Figure 1. The linked database runs under Microsoft Windows 2000 Server Operating System on an Intel(R) XEON(TM) 2.20GHz-based CPU system with 2048 Mbytes of RAM and 471101 Mbytes of total hard disk space. The system has a redundant IBM Workstation/Server with an external tape backup subsystem. Physically, the servers are protected by both key-card access as well as key access and monitored by security camera to maintain personal health information in a manner that is compliant with Health Insurance Portability and Accountability Act of 1996 (HIPAA) standards.

When using LLDB systems for clinical and epidemiologic research, numerous options exist for performing queries to identify patients with a diagnosis of interest. Queries can be based on different sources of diagnostic information, different query strategies or combinations of sources and search strategies (Rector et al. 2004). In the future, additional modification to search strategies utilizing a method based on the hidden Markov chain may facilitate searching genomic databases (Smith et al. 2003). The availability of diagnosis data from numerous sources accentuates the need to ascertain the best method for identifying patients with a specific histologic diagnosis, since these sources can potentially yield different results depending on the query. The linked Medicare-SEER database represents a large linked administrative dataset that is frequently used in clinical and epidemiologic research. Several studies have examined strategies to identify diagnoses of interest with a focus on the use of ICD-9 codes. Many of these studies conclude that case-identification strategie**s** based on ICD-9 codes remain inadequate (Barzilai et al. 2004, Cooper et al. 1999a, Rolnick et al. 2004, Warren et al. 1999).

We designed and tested database search strategies to identify a cohort of patients with follicular lymphoma using the above-mentioned heterogeneous data sources. While other investigators have examined strategies for identifying patients with cancer at a particular site (Nattinger et al. 2004; Rolnick et al. 2004; Warren et al. 1999), cases of follicular lymphoma were selected as a suitable study population since this represents a histologic diagnosis that is important to distinguish from other forms of non-Hodgkin lymphoma and frequently can be misclassified in administrative datasets. The ability of each search strategy to correctly identify patients with follicular lymphoma was examined to determine the most sensitive and specific search strategy. The aim of this study was to use follicular lymphoma as a challenging diagnosis to identify with computer search methods in order to determine ‘best practices’ recommendations for developing search strategies in cancer-focused LLDB systems, such as SEER linked to administrative datasets.

## Methods

### Queries

We utilized a series of search strategies to identify a joint population of interest containing potential patients with follicular lymphoma and then sought to ascertain their histologic diagnosis by reviewing pathology reports. An initial population originated from a list of 817 patients supplied from the Emory University Cancer Registry database (MRS Cancer Registry, IMPAC Medical Systems, Inc., Cambridge, MA). This source provides SEER data for the Atlanta registry. The population was derived from an existing list of non-Hodgkin lymphoma patients from 1985–2002. We used the social security number as the patient identifier. A data scrubbing process using the social security number was performed to obtain the medical record numbers needed to query GeneSys SI. This reduced the list to 783 patients of whom 425 were found in the LLDB. This population (labeled QCR in Table 2) was selected to enrich the final population with cases of follicular lymphoma in the event that all query strategies yielded few patients with this diagnosis.

In our first query, we searched the LLDB using cancer registry histology codes to identify follicular lymphoma patients. The following SEER ICD-O morphology codes were selected: 9690 (follicular lymphoma, NOS), 9695 (follicular lymphoma, grade 1), 9691(follicular lymphoma, grade 2), 9698 (follicular lymphoma, grade 3) (Percy et al. 2000). The query also included the ICD-O behavior code 3 (malignant neoplasms, primary). This query is labeled Q1 in Tables and Figures.

The next series of queries involved text searches of the electronic medical records. Each text string search was conducted twice, once limited to anatomical pathology (AP) reports, and once accessing all medical records. The electronic medical records of a sample of the Q1 population were examined to develop a list of text string candidates. The phrase ‘follicular lymphoma’ was determined to be the most promising phrase. To support our aims to establish sensitive search strategies, a query using the UMLS Metathesaurus Concept Search was performed to obtain synonyms for follicular lymphoma (2006). This revealed 51 synonyms for follicular lymphoma. We identified 21 terms that had histologic overlap with the World Health Organization definition for follicular lymphoma and would not have been included by other queries (e.g. “Malignant lymphoma, centroblastic-centrocytic, follicular” would have been found by the text query “follicular” NEAR “lymphoma”). Ultimately, five phrases from the UMLS synonym list were incorporated into two queries. Refer to Table 3 for a list of the synonym phrases examined and the final content of the queries from this list. Due to observed variations in the appearance of the words ‘follicular’ and ‘lymphoma’, documents were searched for a) the occurrence of the phrase ‘follicular lymphoma’ and b) the occurrence of the word ‘follicular’ near the word ‘lymphoma’. The NEAR function was used to search for each term using a fixed algorithm of searching within 50 words in either direction of the other term. The six text string document searches are labeled queries Q2, Q3, Q4, Q5, Q11 and Q12.

ICD-9 diagnosis codes in the linked database system were supplied by the administrative systems for the Emory University Hospitals and The Emory Clinic. The Emory University Hospitals utilize the HealthQuest system (McKeeson Information Solutions, Inc., Alpharetta, GA) while The Emory Clinic uses the IDX system (IDX Systems Corporation, Burlington, VT). The potential ICD-9 codes that could be utilized in coding follicular lymphoma, including both unspecified and site-specific disease, include ten codes in the range of 202.0–202.08. The query strategy using ICD-9 codes from the clinic was labeled Q6 and that using ICD-9 codes derived from the hospital system was labeled Q7.

In an effort to define search strategies with improved sensitivity, combination search strategies were designed. Joining Q2 and Q6 utilized a combination of a medical records strategy and an administrative query (Q8). Joining Q4 and Q6 accomplished the same goal with the broader (and potentially more sensitive) search of all medical records (Q9). Combining queries of cancer registry and free text of pathology reports with specified terms (believed to be the two most specific strategies a priori) was performed to establish a highly specific and highly sensitive search strategy.

### Confirmation of histiologic diagnosis

To confirm a diagnosis of follicular lymphoma, the medical records of all patients were examined. For each patient, pathology reports were reviewed to confirm histologic cancer diagnosis. When pathology reports were unable to confirm or refute a diagnosis of follicular lymphoma, the electronic medical record was reviewed to identify other chart evidence (e.g. physician notes) to confirm a diagnosis, which could result in a chart-verified diagnosis of follicular lymphoma. Diagnosis confirmation was complicated by non-uniform terminology on pathology reports resulting from the variation that has existed in lymphoma classification strategies over the past 20 years (Mauch et al. 2004). In all cases, World Health Organization (WHO) classification schema for non-Hodgkin lymphoma was utilized as the gold standard for diagnosis (Jaffe et al. 2001). A hematological oncologist (CF) resolved all cases where there was uncertainty as to whether the WHO criteria for follicular lymphoma were met. The disease-verified status was then used to calculate the sensitivity and specificity of each query strategy for detecting this histologic diagnosis in the LLDB. The total population of 1538 patients found through the 13 queries was used in the calculations of sensitivity and specificity. A receiver-operator plot was constructed to compare characteristics of the search strategies.

## Results

The first query based on cancer registry histology codes (Q1) returned 242 patients. Searching pathology reports for the terms ‘follicular’ and ‘lymphoma’ yielded 406 patients when the NEAR operator was used (Q2) and 126 patients when a text string was chosen (Q3). Free text searches of all medical records using the same search strategies found 531 patients with the use of the NEAR operator (Q4) and 193 patients when the terms were combined (Q5). The queries using additional phrases from the UMLS synonym list retrieved relatively few patients (36 and 121 for Q11 and Q12, respectively), only 18 of whom were unique to the entire study population of 1538. Nine hundred and one patients were found associated with potential ICD-9 codes for follicular lymphoma (Q6) from electronic medical records. A smaller group of 288 patients was retrieved with these ICD-9 codes from hospital diagnosis records (Q7). Combining results from Q2 and Q6 retrieved 1137 patients (Q8). Combining populations from Q4 and Q6 yielded 1233 patients (Q9). A combination of results in queries Q1 and Q2 gave a combined population of 498 (Q10). Together the thirteen query populations resulted in 1538 unique patients. All query results are summarized in Table 2.

**Figure 2.**
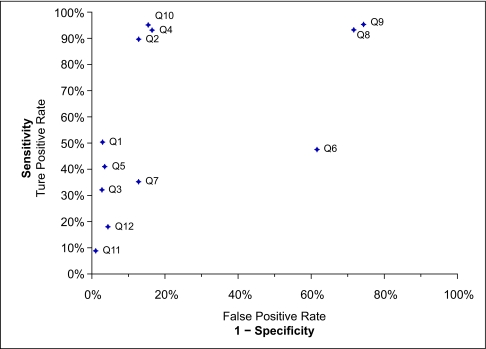
Receiver-operator plot of query strategies to identify a pathology-confirmed histologic diagnosis of follicular lymphoma

The results of disease confirmation, sensitivity, and specificity for each query strategy are summarized in Table 4. Queries that utilized SEER histology codes (Q1), text searches of electronic medical record reports for the term ‘follicular lymphoma’ (Q3, Q5), and terms from the UMLS synonym list (Q11, Q12) had the greatest pathological-confirmed specificity, 97.4%, 96.5%, 99.0% and 95.7% respectively. Query strategies that used the NEAR operator in free-text searches (Q2, Q4, Q8, Q9, Q10) had higher sensitivity for identifying cases of follicular lymphoma, 89.7%, 93.0%, 93.3%, 95.3%, and 95.0% respectively. Queries using free-text searches of pathology records (Q2, Q3) identified fewer cases of follicular lymphoma without marked improvements in specificity when compared with similar free-text searches of all medical records (Q4, Q5). The queries using the NEAR operator in free-text searches alone (Q2, Q4) or in combination with cancer registry histology codes (Q10) yielded the most favorable search strategy characteristics. False positive results commonly occurred in free text searches due to the inclusion of the phrase of interesting in text discussing a differential diagnosis or diagnosis that had been ruled out. A receiver-operator plot (Figure 2) shows an upper-left quadrant clustering of queries Q2, Q4, and Q10 representing those that simultaneously maximized sensitivity and specificity. Combining SEER ICD-O histology codes with a free text search of pathology reports using the NEAR operator provided the most favorable characteristics with a sensitivity of 95% and a specificity of 85% and identified 337 of 415 cases of follicular lymphoma present in this dataset.

## Discussion

We examined a series of query strategies designed to identify patients with a histologic diagnosis of follicular lymphoma in a cancer information system composed of linked, heterogeneous, legacy databases. Our findings indicate that free-text search strategies of electronic medical records and subpopulations of the medical record, such as pathology notes, can provide accurate methods to identify patients with a histologic cancer diagnosis. These query strategies were comparable to a query on coded entries for cancer histology by ICD-O codes in the linked subset of the Emory University cancer registry, a source dataset for the Atlanta SEER database.

Although this study is limited by its focus on a single disease entity, our results suggest that free-text searches of electronic medical records can provide an accurate means of identifying populations of interest. Text searches of electronic medical records may allow for greater accuracy for disease identification but require experimentation to determine the best search strings to employ. A search of the UMLS Knowledge Source Server can be performed to ensure that additional possibilities for describing a particular disease are included in the text string search. Searches using UMLS-derived phrases other than “follicular lymphoma”, while highly specific, identified few additional patients. Other coded medical vocabularies potentially may provide more accurate means for identifying sub-populations with a particular pathological diagnosis, but these are rarely present in legacy data systems where the majority of patient data exists. However, terms from vocabularies such as the Systematized Nomenclature of Medicine (SNOMED) or the Medical Entities Dictionary (MED) could also be employed to identify additional terms for free-text searches.

Queries that utilized the free-text search strategy for ‘follicular lymphoma’ in across all document had high sensitivity and specificity likely due to the mention of this term in chart notes and pathology reports for these patients. When examining the six queries of document searches, limiting the search to pathology reports appears to have marginally improved specificity. Similarly, searching the phrase ‘follicular lymphoma’ was more accurate than using the NEAR function to search for ‘follicular’ within a fixed distance of 50 words from the word ‘lymphoma’. These broader searches increase sensitivity but decrease specificity. Future modifications to the free-text search strategies such as varying the proximity parameter for the NEAR operator or allowing for fuzzy matching may continue to improve this methodology. Our results also indicate that combination queries tend to increase sensitivity at the expense of lowering specificity. However, judicious use of combination queries may allow for expansion of cohort populations with limited effects on specificity.

Search strategies that utilized cancer registry diagnosis information (ICD-O codes) yield similar sensitivity and specificity as that of text searches of electronic medical records, but identify fewer overall cancer cases. This is likely due to the high degree of coding accuracy of the cancer registrars, and the presence of patients with follicular lymphoma treated at the cancer center that did not meet criteria for entry in the registry. In ongoing research studies on prostate cancer (Graiser et al. 2005c) and hepatocellular carcinoma (Graiser et al. 2005a), we also observed that cancer registry codes and structured free-text queries provide improved means for identifying subpopulations of patients with a particular cancer diagnosis. However, cancer registry diagnostic data provide a more efficient source for obtaining accurate patient disease identification.

The ICD-O is used broadly in United States by cancer registry systems including SEER for coding the site (topography) and the histology (morphology) of neoplasms, with a separate one-digit code provided for histologic grading or differentiation. The ICD-O has been published English, Flemish/Dutch, German, Japanese, Korean, Romanian, and Turkish and has translations in development for several other languages. In contrast, ICD-9 codes, which are used extensively in health care databases, typically mix description of the site and type of neoplasm. Moreover, the greater accuracy in ICD-O over ICD-9 codes may also be due to the fact that the cancer registry data is annotated and entered by professionals abstracting patient cases from a thorough review of the patient’s medical records as compared to ICD-9 codes that may be entered by billing clerks who may not collect these additional data. However, reliance on ICD-O codes may produce inaccuracies due changes in disease classification schema over time, inter-observer differences in classification, and may provide incomplete information on cancer morphology, sub-site, and behavior. (Clarke et al. 2004; Glaser et al. 2001; Castillo et al. 2004; Patriarca et al. 2001) Nevertheless, for complex diagnostic entities like the non-Hodgkin lymphomas, ICD-O codes currently provide the best means in common practice for classifying clinically-relevant, histologic subsets of cancer. ICD-O codes also remain the basis for estimating population trends in cancer incidence and identifying new risk factors for cancer (Groves et al. 2000; Morton et al. 2006).

As seen in our study, query strategies based on ICD-O codes are more useful than searches based on ICD-9 codes. This is a reasonable search strategy for cancer-related LLDBs since most cancer registry systems, including the SEER database, already collect these data. Although not reported externally, many health care systems also have internal tumor registries that collect ICD-O codes in their database. Despite the availability of ICD-O codes in cancer registry databases, most clinical and epidemiologic studies using LLDBs continue to rely on ICD-9 diagnostic codes.

Previous studies utilizing linked databases, including Medicare, Medicaid, SEER, HMO, and other administrative sources, have evaluated the use of ICD-9 diagnostic codes for case identification. Most of these studies have found significant discordance between ICD-9 diagnosis codes from Medicare claims and cancer registry data (McClish et al. 1997; Benesch et al. 1997; Schrag et al. 2002). Our study confirms the low sensitivity and specificity of ICD-9 diagnosis codes for providing precise histologic diagnosis information, and highlights the need for more accurate means of case identification if LLDBs are to be used for outcomes research. Moreover, our findings validate the findings of epidemiological studies based on ICD-O diagnoses (Groves et al. 2000; Morton et al. 2006), and corroborate other researchers who have challenged the use of ICD-9 codes for cancer outcomes research (Koroukian et al. 2003; McClish et al. 1997; Warren et al. 2002).

## Conclusion

As electronic medical records systems and methods of linking these systems to other clinical and administrative databases become more widespread, developing methods to utilize these linkages for clinical and epidemiologic research will become increasingly important (Cooper et al. 1999b). Currently, large-linked databases containing patient-specific administrative data are used for cancer outcomes research and bioinformatics research. Clearly delineated methods for identifying subjects with a histologic diagnosis of cancer are needed in order for biologically-relevant conclusions to be drawn from analyses of these data. Moreover, as linked-legacy databases are increasingly being used to by academic centers to identify patients for cancer biomarker studies, biologically-targeted therapies, genomics, and other research endeavors, methods to identify patients with a histologic diagnosis rather than a clinical diagnosis become even more important. Our work provides a first step toward this aim, utilizing a challenging histologic diagnosis that is often misclassified in clinical and administrative datasets. Future research using linked-cancer databases for studies that focus on a population with a precise histologic diagnosis may benefit from case identification procedures that are based on ICD-O or include structured free text search strategies. Additional studies are ongoing to confirm these findings for other cancer diagnoses.

## Figures and Tables

**Table 1.** GeneSys SI sources databases and start dates for source data.

**DATA SOURCE**	**STARTING DATE**
DATA WAREHOUSE	
Hospital administrative (HealthQuest)	1995
Clinic administrative (IDX)	1994
Medical Records	1987
Clinical Labs	2001
Hospital Pharmacy	1998
Clinic Pharmacy	2002
CANCER REGISTRY	
Emory Hospital	1977
Crawford Long Hospital	1981
CLINICAL TRIALS	1981
ELECTRONIC MEDICAL	1991
RECORD (Cerner PowerChart)	
RADIATION ONCOLOGY	
The Emory Clinic	1994
Crawford Long Hospital	2001
GENOMICS (data structures)	2004
FORMS (e.g. informed consent)	2003

**Table 2.** Queries to identify follicular lymphoma cases within a linked legacy database.

**QUERY**	**SOURCE**	**CRITERIA**	**RECORDS IDENTIFIED (% of all records)**
QCR	Imported list from Cancer Registry	Follicular lymphoma by histology codes, 1985–2002	425 (28%)
Q1	Cancer Registry, ICD-O morphology codes plus behavior code 3 (malignant, primary)	Morphology codes 9690, 9691, 9695, 9698 plus behavior code 3	242 (16%)
Q2	Text search–pathology reports	‘follicular’ NEAR ‘lymphoma’	406 (26%)
Q3	Text search–pathology reports	‘follicular lymphoma’	126 (8%)
Q4	Text search–all medical record reports	‘follicular’ NEAR ‘lymphoma’	531 (35%)
Q5	Test search–all medical record reports	‘follicular lymphoma’	193 (13%)
Q6	ICD-9 codes–Emory Clinic	202.0, 202.00, 202.01, 202.02, 202.03, 202.04, 202.05, 202.06, 202.07, 202.08	901 (59%)
Q7	ICD-9 codes–Emory Hospitals	(same as Q6 above)	288 (19%)
Q8	Q2 + Q6	(see criteria for Q2 and Q6 above)	1137 (74%)
Q9	Q4 + Q6	(see criteria for Q4 and Q6 above)	1233 (80%)
Q10	Q1 + Q2	(see criteria for Q1 and Q2 above)	498 (32%)
Q11	Text search–pathology reports	(UMLS terms–see Table 3)	36 (2%)
Q12	Text search–all medical record reports	(UMLS terms–see Table 3)	121 (8%)
	Total cases reviewed combining all queries		1538

**Table 3.** Description of text queries based on UMLS synonyms for follicular lymphoma.

**Selected UMLS synonyms**	**RECORDS IDENTIFIED (% of all records reviewed)**
“nodular lymphoma”	75 (5%)
“Brill–Symmers”	0
“Brill–Symmers”	0
“reticulosarcoma–follicular”	0
“reticulosarcoma–nodular”	0
“follicular lymphosarcoma”	0
“giant follicular lymphoma”	0
“mal.lym,centr-blas/cyt,foll”	0
“malig.lymphoma, nodular”	0
“malig. lymphoma, nodular”	0
“lymphoma, nodular”	65 (4%)
“nodular lymphosarcoma”	0
“follicle center lymphoma”	13 (<1%)
“follicular non-Hodgkin”	35 (2.3%)
“foll low grade B-cell lymphoma”	0
“germinoblastoma, follicular”	0
“lymphoma, follicle center”	4 (<1%)
“malignant lymphoma, lymphocytic, nodular”	0
“lyoma,centrbl-centrcyt,foll”	0
“reticulosarcoma, follicular”	0
“reticulosarcoma, nodular”	0

**Table 4.** Sensitivity and specificity for linked database queries.

**Query**	**True Positive**	**False Positive**	**True Negative**	**False negative**	**Sensitivity (%)**		**Specificity (%)**	
					**Path**	**All**	**Path**	**All**
Q1	151 + *44* = 195	23 + *24* = 47	772 + *304* = 1076	149 + *71* = 220	50.3	47.0	97.1	95.8
Q2	269 + *19* = 288	102 + *16* = 118	693 + *312* = 1004	31 + *96* = 127	89.7	69.4	87.2	89.5
Q3	96 + *6* = 102	21 + *3* = 24	774 + *325* = 1099	204 + *109* = 313	32.0	24.6	97.4	97.9
Q4	279 + *94* = 373	131 + *27* = 158	664 + *301* = 965	21 + *21* = 42	93.0	90.0	83.5	85.9
Q5	123 + *36* = 159	28 + *6* = 34	767 + *322* = 1089	177 + *79* = 256	41.0	38.3	96.5	97.0
Q6	143 + *35* = 178	490 + *233* = 723	305 + *95* = 400	157 + *80* = 237	47.7	42.9	38.4	35.6
Q7	106 + *31* = 137	101 + *50* = 151	694 + *278* = 972	194 + *84* = 278	35.3	33.0	87.3	86.6
Q8	280 + *43* = 323	569 + *245* = 814	226 + *83* = 309	20 + *72* = 92	93.3	77.8	28.4	27.5
Q9	286 + *102* = 388	591 + *254* = 845	204 + *74* = 278	14 + *13* = 27	95.3	93.5	25.7	24.8
Q10	285 + *52* = 337	123 + *38* = 161	672 + *290* = 962	15 + *63* = 78	95.0	81.2	84.5	85.7
Q11	27 + *1* = 28	8 + *0* = 8	787 + *328*= 1115	273 + *114* = 387	9.0	6.7	99.0	99.3
Q12	54 + *29* = 83	34 + *4* = 38	761 + *324* = 1085	246 + *86* = 332	18.0	20.0	95.7	96.6

**Note:** Each total has a pathology-verified component listed first followed by a chart-verified component in italics.

## Abbreviations:

LLDB: Large Linked Database
SEER: Surveillance Epidemiology and End Results
EMR: Electronic Medical Record
ICD-9: International Classification of Diseases (9th revision)
ICD-O: International Classification of Diseases for Oncology
AP: Anatomical Pathology
WHO: World Health Organization
